# Prediction of latent tuberculosis infection in Venezuelan immigrants: construction and validation of a surveillance model

**DOI:** 10.1590/0037-8682-0434-2024

**Published:** 2025-09-22

**Authors:** Fernanda Zambonin, Nilson Cavalcante de Souza, Elvira Maria Godinho de Seixas Maciel, José Ueleres Braga

**Affiliations:** 1 Fundação Oswaldo Cruz, Escola Nacional de Saúde Pública Sergio Arouca, Rio de Janeiro, RJ, Brasil.; 2 Exército Brasileiro, Operação Acolhida, Boa Vista, RR, Brasil.; 3 Instituto de Medicina Social, Universidade do Estado do Rio de Janeiro, Rio de Janeiro, RJ, Brasil.

**Keywords:** Human migration, Latent tuberculosis, Population dynamics, Public health surveillance

## Abstract

**Background:**

Latent tuberculosis infection (LTBI) is a significant concern among migrant populations, particularly Venezuelans, due to its adverse health and social conditions. This study aimed to construct and validate a predictive model of LTBI among Venezuelan migrants.

**Methods:**

This cross-sectional study utilized data from the project "TB and migrants in BRICS countries: The case of Brazil”, carried out in Boa Vista, Roraima, in 2020. The final sample included 427 participants. For the analysis, 22 variables were selected, and simple and multiple logistic regression analyses were applied. General measures (Nagalkerke's R^2^ and Brier’s score), discriminative capacity (accuracy, receiver operating characteristic curve, and area under the curve [AUC]), and calibration measures (Hosmer-Lemeshow test and calibration graph) were used to evaluate the model. The model was internally validated using bootstrapping. Finally, a nomogram and a clinical decision curve were constructed.

**Results:**

Six LTBI predictors (marital status, social benefit, documentation status, smoking status, presence of comorbidities, and fever) were included in the final model. The predictive model demonstrated moderate discriminatory capacity (AUC: 0.676), good calibration, and was also validated with an AUC of 0.678. Additionally, a clinical decision analysis revealed that the use of the model offers superior benefits compared with traditional treatment strategies.

**Conclusions:**

The predictive model and nomogram proved to be useful tools for LTBI screening in migrants, potentially guiding border health surveillance actions in this population.

## INTRODUCTION

Tuberculosis (TB) remains a global public health concern. In 2022, 7.5 million new cases will be diagnosed, the highest number recorded since the World Health Organization began global monitoring of the disease in 1995^1^ ^,^ ^2^. In response to this, a key target of the World Health Organization’s “End TB” strategy is to achieve an 80% reduction in tuberculosis incidence by 2030 compared with 2015 levels^3^, an objective that requires coordinated and sustained efforts to be achieved.

Historically, TB control programs focused on the management and control of active TB infections. However, as research has progressed, it has become clear that this approach alone is not sufficient to eradicate the disease. The elimination of TB also depends on the treatment of infection reservoirs, that is, latent tuberculosis infection (LTBI)^4^.

In this context, LTBI management has become an essential component of TB elimination. Identifying and treating individuals at the highest risk of developing active disease is particularly crucial^5^ ^,^ ^6^, aligning with the 'integrated, patient-centered care and prevention' pillar of the ‘End TB’ strategy, which recommends preventive treatment for individuals at high risk of TB^7^. 

Because not the entire population benefits from screening for LTBI, the use of predictive models has emerged as a valuable tool for identifying individuals at a higher risk of developing the disease. These models help estimate the likelihood of a person having or developing TB, allowing clinical decisions to be based on robust data, and directing resources to those most at risk^8^. Thus, predictive models optimize public health strategies by focusing on groups with the greatest potential for benefits.

Migrants are particularly vulnerable to infectious diseases such as TB. Several factors along the migratory trajectory can influence this risk. In the pre-migratory phase, the prevalence of infectious diseases in the country of origin and access to preventive care, such as vaccines, are critical aspects. Transport conditions, overcrowding, and prolonged exposure to unhealthy environments also increase the risk. In the post-migration phase, social determinants such as inadequate housing, poor working conditions, and limited access to healthcare-alongside individual factors like comorbidities and immune status-contribute significantly to increased vulnerability to TB^9^. 

A narrative review evaluated the effectiveness and cost-effectiveness of LTBI screening in migrants and concluded that this strategy should be aimed at high-risk groups, such as migrants from countries with a high TB burden, to achieve better results^10^. A different review that analyzed screening policies for TB and LTBI in migrants in Europe highlighted the need to harmonize case definitions and screening strategies, given the diversity of approaches among countries^11^.

Given the scarcity of predictive models targeting migrant populations at increased risk of LTBI, and those most likely to benefit from screening, this study aimed to address this gap by focusing screening efforts on Venezuelan migrants entering Brazil.. Therefore, the objective of the present research is to build and validate a predictive model for LTBI in Venezuelan migrants.

## METHODS

The present study is a cross-sectional study based on secondary data from the project “TB and migrants in BRICS countries: the case of Brazil”, carried out in Boa Vista, Roraima, Brazil, in 2020. The initial sample included 458 individuals, of whom 31 (6.8%) were excluded because of missing data on the selected variables, resulting in a final sample of 427 participants.

Of the 203 variables available in the original database, 22 were selected for the analysis. The outcome variable was reactivity to the tuberculin skin test (TST), and the predictor variables were categorized into sociodemographic factors (sex, race, age, marital status, years of study, occupation, receiving social benefit, and type of housing), factors related to migration (time of migration, reason for migration, documentary status, and time spent in Brazil), health history (BCG scar, alcohol use, smoking, comorbidities, history of TB, and contact with TB), and clinical data (cough, fever, weight loss, and sweating).

Binary logistic regression was used to identify the predictors of LTBI. The initial selection of predictors was performed by simple logistic regression, with variables having p-values less than 20%, and those considered theoretically relevant were included in the multiple regression. Subsequently, the stepwise regression (backward stepwise) technique was applied, beginning with a saturated model and progressively removing the least significant predictors based on likelihood ratio tests and p-values. The significance level adopted was 5%.

Possible violations of the model assumptions, such as error independence and multicollinearity, were evaluated, with the latter verified by the variance inflation factor (VIF) (VIF > 10). The final model was chosen based on maximum likelihood and the Akaike information criterion (AIC). For the predictors selected in the final model, odds ratios and their respective 95% confidence intervals were calculated.

The performance of the model was evaluated using Nagelkerke's R2, Brier’s score, ROC curve, and the area under the curve (AUC). Calibration was verified using a calibration chart and the Hosmer-Lemeshow test. Internal validation was performed using the bootstrap method with resampling of at least 100 subjects.

Additionally, a nomogram was constructed to present the predictive model, and a clinical decision curve was used to evaluate its clinical utility. All analyses were conducted using R software, and the study was approved by the Ethics and Research Committee of the National School of Public Health (ENSP/Fiocruz) under CAAE No. 77525924.1.0000.5240.

## RESULTS

In the present study, 104 individuals showed reactivity in the test (TST), indicating a prevalence of 24.36 per 100 migrants tested. Table 1 presents the descriptive analysis and univariate binary logistic regression of the biological, socioeconomic, migration-related, health history, and clinical predictors of LTBI in Venezuelan migrants living in Boa Vista, Roraima, Brazil, in 2020. 

**TABLE 1:** Univariate logistic regression of LTBI in Venezuelan migrants living in Boa Vista, Roraima in 2020. Boa Vista, Roraima, Brazil 2024.

Variable	n (%)	Reactivity to TST		p-value
		No (n=323)	Yes (n=104)	
		n (%)	n (%)	
**Block 1- Biological and socioeconomic predictors**				
**Gender**				
Female	251 (58.8)	197 (61.0)	54 (51.9)	0.103*
Male	176 (41.2)	126 (39)	50 (48.1)	
**Race**				
White	102 (23.9)	79 (24.5)	23 (22.1)	0.316
Brown/Yellow	208 (48.7)	159 (49.2)	49 (47.1)	
Black	90 (21.1)	64 (19.8)	26 (25.0)	
Other	27 (6.3)	21 ( 6.5)	6 ( 5.8)	
**Age (Mean; ±)**	36.18 (13.74)	35.61 (13.39)	37.93 (14.72)	0.135
**Marital Status**				
Single	237 (55.5)	174 (53.9)	63 (60.6)	0.019*
Married common law union	165 (38.6)	134 (41.5)	31 (29.8)	
Widowed/divorced	25 (5.9)	15 (4.6)	10 (9.6)	
**Years of study**				
Up to 4 years	47 (11)	36 (11.1)	11 (10.6)	0.356
Between 5 and 8 years	140 (32.8)	102 (31.6)	38 (36.5)	
9 years and over	240 (56.2)	185 (57.3)	55 (52.9)	
**Occupation**				
Formal worker	22 (5.2)	15 (4.6)	7 (6.7)	0.475
Informal worker	98 (23)	70 (21.7)	28 (26.9)	
Unemployed	275 (64.4)	210 (65)	65 (62.5)	
Retired	6 (1.4)	5 (1.5)	1 (1)	
Other	26 (6)	23 (7.1)	3 (2.9)	
**Receives social benefit**				
Yes	306 (71.7)	239 (74)	67 (64.4)	0.061*
No	121 (28.3)	84 (26)	37 (35.6)	
**Housing**				
Rented house	10 (2.3)	5 (1.5)	5 (4.8)	0.070*
Own house	0	0 (0.0)	0 (0.0)	
Student asylum	2 (0.5)	1 (1.3)	1 (1.0)	
In asylum/shelter/hostel	414 (97)	316 (97.8)	98 (94.2)	
Other	1 (0.2)	1 (0.3)	0 (0.0)	
**Block 2 - Predictors related to migration**				
Migration time				
Less than 6 months	32 (7.5)	26 (8)	6 (5.8)	0.402
6 months to <2 years	323 (75.6)	244 (75.5)	79 (76)	
≥ 2 years	72 (16.9)	53 (16.4)	19 (18.3)	
**Reason for migration**				
Vulnerability in the country of origin	251 (58.8)	196 (60.7)	55 (52.9)	0.185*
Work	92 (21.5)	72 (22.3)	20 (19.2)	
Health	57 (13.3)	37 (11.5)	20 (19.2)	
Other	27 (6.3)	18 (5.6)	9 (8.7)	
**Documentary status**				
Regular	359 (84.1)	267 (82.7)	92 (88.5)	0.162*
Irregular	44 (10.3)	37 (11.5)	7 (6.7)	
In the process of regularization	24 (5.6)	19 (5.9)	5 (4.8)	
**Length of time intended to live in Brazil**				
Improvement of crises situation in migrants’ country of origin	159 (37.2)	128 (39.6)	31 (29.8)	0.053*
Permanently	235 (55)	169 (52.3)	66 (63.5)	
Other	33 (7.7)	26 (8)	7 (6.7)	
**Block 3- Predictors related to health history**				
**BGC scar**				
No	63 (14.8)	52 (16.1)	11 (10.6)	0.170*
Yes	364 (85.2)	271 (83.9)	93 (89.4)	
**Alcohol use**				
Does not consume	332 (77.8)	253 (78.3)	79 (76)	0.343
One day a week	50 (11.7)	35 (10.8)	15 (14.4)	
Two or more days a week	45 (10.5)	35 (10.8)	10 (9.6)	
**Smoking**				
Never smoked	262 (61.4)	208 (64.4)	54 (51.9)	0.007*
Former smoker	45 (10.5)	35 (10.8)	10 (9.6)	
Current smoker	160 (28.1)	80 (24.8)	40 (38.5)	
**Comorbidities**				
No	377 (88.3)	294 (91)	83 (79.8)	0.003*
Yes	50 (11.7)	29 (9)	21 (20.2)	
**History of tuberculosis**				
Yes	4 (0.9)	2 (0.6)	2 (1.9)	0.723
No	414 (97)	315 (97.5)	99 (95.2)	
Does not know	9 (2.1)	6 (1.9)	3 (2.9)	
**Contact with tuberculosis**				
Yes	51 (12.1)	34 (10.7)	17 (16.3)	0.046*
No	342 (80.9)	265 (83.1)	77 (74)	
Does not know	30 (7.1)	20 (6.3)	10 (9.6)	
**Block 4-Clinical predictors**				
Cough (Yes)	31 (7.3)	23 (7.1)	8 (7.8)	0.862
Fever (Yes)	10 (2.3)	5 (1.6)	5 (4.8)	0.081*
Weight loss (Yes)	45 (10.6)	31 (9.6)	14 (13.6)	0.519
Sweating (Yes)	8 (1.9)	7 (2.2)	1 (1.0)	0.437

**Legend:** (*): Significantly associated with the outcome at a significance level of 20% (p-value <0.20); **TST:** tuberculin skin test.

Of the 22 variables collected from the study cohort, 12 were selected at a significance level of 20% (p-value < 0.2). The variables selected to compose the multiple model were sex, marital status, receiving social benefits, type of housing, reason for migration, documentary status, time spent in Brazil, presence of a BCG scar, smoking, comorbidities, contact with someone with tuberculosis, and self-reported fever. 

Selection using the stepwise (backward stepwise) method was applied to develop the predictive model, and only variables with significance levels < 5% remained in the final model, as shown in Table 2. The final model had six variables and was chosen using a likelihood ratio test with the AIC information criterion.

The final model indicated that being widowed or divorced (OR= 2.60; CI: 0.99-6.61; p= 0.0469), not receiving social benefits (OR= 2.28; CI: 1.30-3.9; p= 0.00424), having regular documentation status (OR= 2.74; CI: 1.25-8.16; p= 0.03457), being a current smoker (OR= 2.15; CI: 1.28-3.62; p= 0.00385), having comorbidities (OR= 3.15; CI: 1.59-6.20; p= 0.00086), and self-reported fever (OR= 3.17; CI: 0.84-11.98; p= 0.04098) are predictors associated with a higher risk of LTBI among Venezuelan migrants in Boa Vista, Roraima, Brazil. 

**TABLE 2:** Multiple logistic regression and predictors associated with LTBI in Venezuelan migrants residing in Boa Vista, Roraima, Brazil in 2020. Boa Vista, Roraima, Brazil 2024.

Variable	Levels	Odds ratio (OR) Confidence interval (CI)	p-value
Marital status	Married	1 (reference)	-
	Single	1.46 (0.87-2.48)	0.1570
	Widowed/divorced	2.60 (0.99-6.61)	0.0469*
Receives social benefit	Yes	1 (reference)	-
	No	2.28 (1.30-3.9)	0.00424*
Documental status	Irregular	1 (reference)	-
	In the process of regularization	1.63 (0.41-6.11)	0.47345
	Regular	2.74 (1.25-8.16)	0.03457*
Smoking	Never smoked	1 (reference)	-
	Former smoker	1.10 (0.47-2.40)	0.80701
	Currently smokes	2.15 (1.28-3.62)	0.00385*
Comorbidity	No	1 (reference)	-
	Yes	3.15 (1.59-6.20)	0.00086*
Fever	No	1 (reference)	-
	Yes	3.17 (0.84-11.98)	0.04098*
AIC	442.93	-	-

**Legend:** (*) significantly associated with outcome at a significance level of 5% (p-value <0.05).

To assess model assumptions, multicollinearity among predictor variables was evaluated using the generalized variance inflation factor (GVIF) technique. No variables presented multicollinearity (VIF<10). The predictive model achieved an overall accuracy of 76.94% (95% CI 72.57-80.92), with a sensitivity of 74% and specificity of 52%, based on the confusion matrix at a cutoff point of 0.1805. Model performance metrics included a Nagelkerke’s R² of 0.11 and a Brier score of 0.095.

**FIGURE 1:**
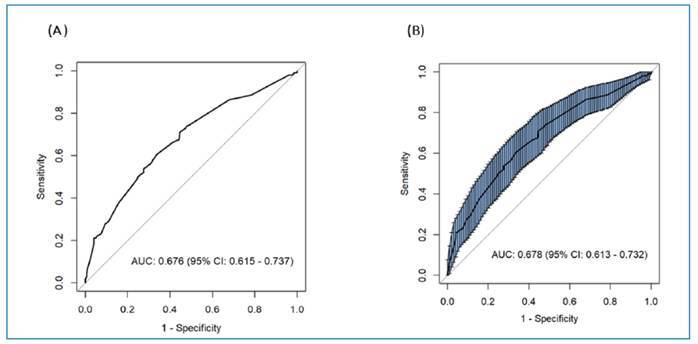
ROC curve for the predictive model and internal validation by the bootstrap method for LTBI in Venezuelan migrants in Boa Vista, Roraima (2020). Boa Vista, Roraima, Brazil 2020. **Legend: (A):** ROC curve and AUC of the final predictive model; **(B):** ROC curve and AUC (CI95%) of internal validation using the bootstrap method; **ROC:** Receiver operating characteristic curve; **AUC:** Area under the curve; **CI:** Confidence interval.

As shown in Figure 1, the AUC for the predictive model was 0.676 (95% CI 0.615-0.737), while the AUC for internal validation using the bootstrap method (resampling 500) was 0.678 (95% CI 0.613-0.732), suggesting that the model had discriminative ability.

**FIGURE 2:**
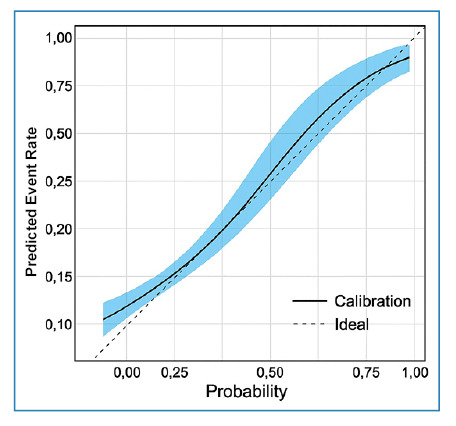
Calibration curve of the predictive model.

Analysis of the calibration curve suggested that the predictive model had acceptable calibration in most probability ranges, as shown in Figure 2. The Hosmer-Lemeshow test produced a non-significant statistical value (p=0.8557), suggesting that there was no deviation from a perfect fit between prediction and observation.

**FIGURE 3:**
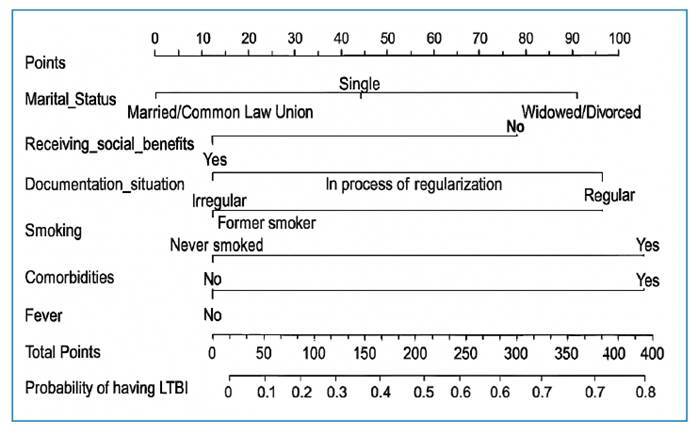
Nomogram for LTBI in Venezuelan migrants and its algorithm. **Legend: LTBI:** Latent tuberculosis infection.

Based on the predictive model, a nomogram was constructed as a quantitative tool for predicting the likelihood of LTBI among Venezuelan migrants (Figure 3).

The decision curve showed that when the estimated probability of LTBI risk in Venezuelan migrants was between 20% and 60%, applying the predictive model to predict household contact TB infection would add more benefits than the treat-all or treat-nothing strategies.

## DISCUSSION

In the present study, the prevalence of LTBI among Venezuelan migrants, measured by TST reactivity, was 24.36%, which is close to the global estimate of 23.0% (95% CI: 20.4% -26.4%), and above the average for the American continent, estimated at 17%^12^. Studies conducted with migrant populations show great variability in the prevalence of LTBI, with rates ranging from 12% in Rome^13^, 18.4% in Mexico^14^, 20.4% in Singapore^15^, to 45% in Malta^16^. 

In Brazil, the flow of migrants has increased significantly in recent years, especially from Venezuela, owing to economic and political crises in neighboring countries. The crisis prompted large-scale migration as the population sought better living conditions in response to the collapse of essential services, including the healthcare system^17^. This structural fragility contributes to the resurgence of infectious diseases that have been previously controlled or eliminated^18^, and Special attention should be paid to TB and LTBI.

A multicenter study conducted in four Brazilian capitals (Boa Vista, Manaus, São Paulo, and Curitiba) among international migrants, of whom 77% were Venezuelans, found a high prevalence of LTBI, ranging from 23.5% to 46.1%^18^.

Differences in prevalence rates can be explained by factors such as the region of origin and destination of migrants, especially when individuals from countries with a high TB burden migrate to nations with a low incidence^19^. However, Brazil and Venezuela had similar incidence rates (49/100 million inhabitants and 45/100 million inhabitants, respectively), both considered high^20^.

Although it is often difficult to identify the place of origin of TB exposure, there is evidence that most cases of active TB among migrants result from reactivation of LTBI acquired before migration^10^ ^,^ ^11^ ^,^ ^21^. Therefore, it is necessary to evaluate not only the characteristics of the countries but also the individual risk for LTBI, considering variables such as migratory route, age, sex, health status, socioeconomic conditions, and reasons for migration^22^. 

From this perspective, predictive models play an important role in directing professionals and health actions to predict the probability of LTBI infection, thus guiding clinical decision-making to obtain a timely diagnosis with an emphasis on the population at greatest risk^23^. According to a literature survey, existing predictive models have been developed for assessing the risk of tuberculosis transmission among contacts^24^, for LTBI screening in health professionals^25^, and for identifying high-risk patients with LTBI prone to active TB^26^. However, no study has predicted LTBI in migrant populations. In addition, not all migrants benefit from screening for LTBI, and the predictive model provides a direction for identifying those at a higher risk. 

The predictive model of the present study incorporated six predictors associated with an increased risk of LTBI in Venezuelan migrants: two sociodemographic factors (marital status and receiving social benefits), one related to migration (documentary status), two related to health status (smoking and the presence of comorbidities), and one clinical predictor (fever). All variables were accessible in a clinical context, which facilitated the practical applicability of the model. 

For a predictive model to be considered for practical application, it must perform well in terms of discriminatory and specific capacities and decision clinical analysis (DCA)^23^ ^,^ ^27^. In the present study, the predictive model demonstrated moderate discriminatory capacity (AUC: 0.676). Internal validation based on the bootstrap method showed a similar result (AUC: 0.678), as well as good calibration capacity. The decision curve analysis reinforced its clinical utility, indicating that the adoption of the model would provide greater benefits than the treatment or treatment strategies. A nomogram was constructed for practical application. 

Divorced and single individuals had a higher risk of LTBI. A study conducted in Singapore found similar results, showing that divorced or separated individuals (OR 2.36; 95% CI 1.38-4.02; p=0.002) were significantly more likely to present positive LTBI (15). Pang et al.^28^ suggest that marital status, especially in singles, widowers, or divorcees, may be related to the absence or low level of family support, which increases vulnerability to diseases such as TB during times of psychosocial stress.

Not receiving social benefits was also considered a significant predictor of LTBI in Venezuelan migrants, suggesting that social vulnerabilities, such as a lack of financial support, may contribute to LTBI risk. The association between poverty and the development of TB has been well proven in scientific literature^29^ ^-^ ^31^, and is considered the root cause of TB^32^. This is because poverty extends beyond monetary deprivation, encompassing overlapping risk factors that make it a multidimensional phenomenon^26^ ^,^ ^28^. Individuals considered poor are exposed to precarious housing and working conditions, greater food insecurity, and difficulty accessing healthcare services^33^, all of which increase the risk of LTBI and TB. 

This situation is further aggravated among migrants, since they often face additional barriers such as a lack of regular documents, discrimination, and limited support networks in host countries. Without adequate financial support, many migrants are exposed to overcrowded and unsanitary living conditions, in addition to facing difficulties in accessing quality healthcare^34^, which are factors that contribute to the risk of LTBI.

Regularized documentation was included as a significant variable in the predictive model of LTBI among Venezuelan migrants. This result may be attributed to the study sample, which primarily consisted of individuals residing in shelters who received support from government agencies to facilitate the regularization of their documentation. Another aspect that must be considered is the barriers migrants face in the host country, especially when accessing health services. 

Although the Brazilian unified healthcare system offers universal care, administrative barriers limit the full use of these services. Bureaucratic practices such as the requirement for identification documents, proof of residence, and other records can hinder access to undocumented or irregular migrants^35^. Thus, it is easier for migrants with regularized documents to overcome administrative barriers, which favors access to health services and consequently increases LTBI diagnosis rates in this population.

Two clinical predictors were associated with a higher risk of LTBI: smoking status and comorbidities. The main comorbidities investigated were human immunodeficiency virus of HIV, diabetes mellitus (DM), and kidney disease. Both smoking and the presence of these comorbidities interfere with the immune system response, altering macrophage responseswith or without a decrease in CD4 + cells, which increases the susceptibility to *Mycobacterium tuberculosis* ^36^ ^,^ ^37^. Co-infection with HIV^37^, patients with DM^38^, and smoking, especially current smoker^39^, are considered risk factors not only for LTBI, but also for the progression of latent TB to the active form.

Self-reported fever associated with LTBI is a less common finding in the predictive model because LTBI is typically asymptomatic, unlike active TB, in which fever symptoms are common. Fever can easily be confused with other infectious diseases, especially in vulnerable populations, such as migrants, where there is high exposure to multiple risk factors and comorbidities. According to Pai et al.^40^, fever, a clinical indicator of active TB, may be lost amid other infectious conditions prevalent in high-burden areas, creating challenges in the correct identification of latent TB. 

Despite these results, the present study has some limitations that should be considered when interpreting the results. First, the sample was primarily composed of migrants who were already sheltered, which may introduce selection bias, as these individuals are likely to have greater access to documentation regularization and health monitoring. This particularity may limit the generalizability of the model to other migrant populations, such as those living in remote areas or lacking institutional support. Second, the variables used in the model were limited by the scope of the original database, preventing the inclusion of more detailed socioeconomic and housing factors such as the number of people in the household, housing conditions, and household income, which could have contributed to a more in-depth analysis of the risks associated with LTBI. 

Moreover, comorbidities were self-reported, which may have underestimated or overestimated the actual prevalence of health conditions such as HIV, diabetes, and kidney disease, increasing the risk of information bias. The absence of formal diagnostic tests may have affected the accuracy of the observed associations. To address these limitations, future studies should include a more diverse sample of migrants, encompassing varied housing conditions and documentation statuses, and incorporate additional variables that more comprehensively reflect the social determinants of health. Furthermore, the collection of more accurate clinical data, including laboratory diagnoses of comorbidities, is crucial to increase the robustness and applicability of the predictive model.

A model for predicting the risk of LTBI in Venezuelan migrants was developed, incorporating six predictors: two sociodemographic factors, one related to migration, two related to health status, and one clinical factor. The predictive model showed a moderate discriminatory capacity and good calibration. The use of a nomogram and clinical decision curve as additional tools makes the model more accessible to healthcare professionals, facilitating its application in the clinical context. 

These findings reinforce the importance of a predictive model specific to migrants, considering the complexity of factors that affect the health of this population. The practical application of this model in in settings characterized by high migratory flow and social vulnerability may enhance early detection and management of LTBI, thereby reducing the risk of progression to active TB, improving disease control among high-risk populations, and supporting health surveillance efforts at border regions.
